# Enhanced grain quality of malt barley (*Hordeum distichon* L.) in response to mixed use of organic compost and mineral nitrogen rates

**DOI:** 10.1371/journal.pone.0343009

**Published:** 2026-02-17

**Authors:** Arega Wole, Amsalu Nebiyu, Getachew Agegnehu, Yenus Ousman

**Affiliations:** 1 College of Agriculture and Environmental Sciences, Department of Plant Sciences, University of Gondar, Gondar, Ethiopia; 2 College of Agriculture and Veterinary Medicine, Department of Horticulture and Plant Sciences, Jimma University, Jimma, Ethiopia; 3 International Crops Research Institute for the Semi-Arid Tropics, Addis Ababa, Ethiopia; University of Duhok, IRAQ

## Abstract

Declining soil fertility status and poor agronomic management practices are major factors of declining quality for malt barley in the Ethiopian highland area, particularly in the study area. To address these major challenges, a two-year (2022−2023) field experiment was conducted in experimental fields in the Welmera district to evaluate the effects of mixed-use mineral N fertilization and compost rates on malt barley quality parameters. A randomized complete block design with factorial arrangements of five N rates (0, 23, 46, 69, and 92 kg ha^-1^) and four compost rates (0, 2.5, 5, and 7.5 t ha^-1^) was tested in three replications. According to the results, both compost and mineral nitrogen fertilizer were significantly influenced thousand-seed weight, protein content, malt extract, beta-glucan content, malt friability and germination energy of malt barley grain, with seasonal variations. Increased mineral N levels enhanced seed weight and grain protein content but reduced malt extract yield and malt friability, while compost improved grain protein content and malt beta-glucan. These influences were improved by organic compost and mineral fertilization, which enhanced multiple quality parameters. The results clearly demonstrated that application of 69 kg N ha^-1^ and 5 t ha^-1^ of compost rate in moderation, which optimized the malt quality parameters, met industry standards without increasing protein concentration or diminishing malt extract yield of malt barley grain. These mixed management approaches not only enhance the quality of malt barley grain for the beer industry but also help soil fertility restoration and guarantee long-term production sustainability for smallholder farmers in the Ethiopian highlands. For robust and wide applicability, subsequent multiple-seasons and multiple-locations studies with additional quality assessments are recommended.

## Introduction

Barley (*Hordeum vulgare* L.) has high genetic diversity with distinct geographic population structure. The Western Fertile Crescent is considered the primary center of diversity and domestication of the barley crop, while several reports most strongly support the Eastern part of the Fertile Crescent (Iran or the Himalayas) as the second primary center of barley diversity and domestication [1]. Barley is believed to have been domesticated from the wild crop in Ethiopia, Morocco, and Tibet, although robust traces are pointing towards the Middle East, particularly in the region that is encompassed by Israel and Jordan [2]. Morocco, Ethiopia, Algeria, Tunisia, and South Africa were the top five countries in Africa producing barley grain with predicted production, area coverage, and productivity of 2.8, 2.4, 0.56, 0.43, and 0.33 million tons; 1.5, 0.96, 0.53, 0.51, and 0.1 million hectares; and 1.87, 2.45, 1.06, 0.84, and 3.5 tons/ha, respectively [3]. In Ethiopia barley ranks fifth in the acreage and production of cereal crop, following tef, maize, wheat and sorghum [4], while accounting for approximately 25% of the continent’s total barley production [5]**.**

Barley is a critically important crop in Ethiopia for grain food, animal feed using straw, malt production (brewing industry), and income generation of smallholder farmers predominantly found in the highlands, where its use is partially embedded in traditional recipes such as *‘Injera,’* and other types of food [6,7]. However, its productivity (2.18t ha^-1^) is very low compared to the world average (2.89 t ha^-1^) and its potential productivity of 6 t ha^-1^ [8]. This due to low soil fertility levels, high soil acidity, insufficient nutrient inputs application, limited application of organic amendments, and poor agronomic management practices, which are major causes of declining yields for malt barley in Ethiopia [8–10]. Due to low production and quality capacity and outdated malting factories, Ethiopia is forced to allocate millions of dollars annually to import malt barley to meet brewery demands at the national level [11].

Numerous malt barley quality parameters influence the suitability of malt barley production for an acceptable range of malt and beer factories’ beer production [12]. The crucial malting barley includes malt extract yield, diastatic power, grain size, and grain protein accumulation [13]. Malt extract yield based on grain parameters such as grain size, shape, hardness, moisture content, dormancy and free from physical impurities, as well as biochemical factors like protein concentration, beta-glucan, starch concentration and enzyme activities [12]. While barley grain is accessibility of for malting purposes, its quality is below standard and does not meet the standards of malting quality. These due to different factors such as the specific variety, soil properties, and applied fertilizer like N fertilization [14].

Nitrogen fertilizer can be considered a main factor in enhancing the production of malt barley, as it directly affects both yield and quality, as supported by multiple studies [9,15–19]. Similarly, different scholars discovered the effect of N fertilization on different quality parameters of malt barley grain such as grain protein concentration [20–22], malt extract yield [23], malt β-glucan [24], malt friability [25], hectoliter weight [22] and germination capacity [26]. However, the continuous utilization of artificial fertilizer sources results in SOM decline and decreased soil quality of agricultural fields, and its high dose affects microbial activity, leads to stunted plant growth, and reduces soil fertility [27] and also influences the agricultural production, which leads to reduced returns [28], which influences the production and productivity of crop plants. Similarly, continuous reliance on mineral fertilizers, the high cost, and incorporation with organic fertilizer sources create major issues by adversely impacting the soil nutrient availability, quality, and soil structure [29]**.**

Compost is more effective in increasing barley yield and its components, such as grain weight, straw weight, and total yield, and nutrient uptake [30,31]. Additionally, it plays a significant role in improving soil physical and chemical properties [32,33]. Moreover, **t**heir easy accessibility, low cost, and eco-friendly nature make them an advisable choice for enhancing soil fertility and safeguarding the environment [34,35]. Even if organic compost has numerous merits, many farmers are rejecting utilizing it alone due to its slow-release fertilizers, and labor intensive [36–38].

Combined application of organic and mineral amendments can help address the above problems and improve soil properties, ultimately enhancing crop yield as well as quality and finally increasing the economic benefits of malt barley production [10,13]. Although extensive international research has been conducted on mineral N management in barley production, relatively little attention has been given to combined approaches that conjointly use natural and mineral N fertilizer sources under Ethiopian condition. This knowledge gap is particularly critical in the Ethiopian highlands, where compost can provide multiple advantages such as gradual nutrient release, improved soil structure, and restoration of soil organic matter, yet its potential role in enhancing malt barley quality parameters remains unclear. To address this research gap, the current experiment investigates the integrated application of compost and chemical nitrogen to optimize malt barley quality in the Ethiopian highlands, particularly in the study area, while balancing the need for higher yield with the quality standards of the brewing industries. The specific objectives were to (1) evaluate the effects of the integrated use of nitrogen and compost fertilizer rate on malting quality traits and (2) identify the optimum rate of integrated use of mineral nitrogen and compost fertilizer rate that meets the malt quality standard for the malt barley growing area of the Ethiopian highlands.

## Materials and methods

### Description of the study area

**Fig 1:**
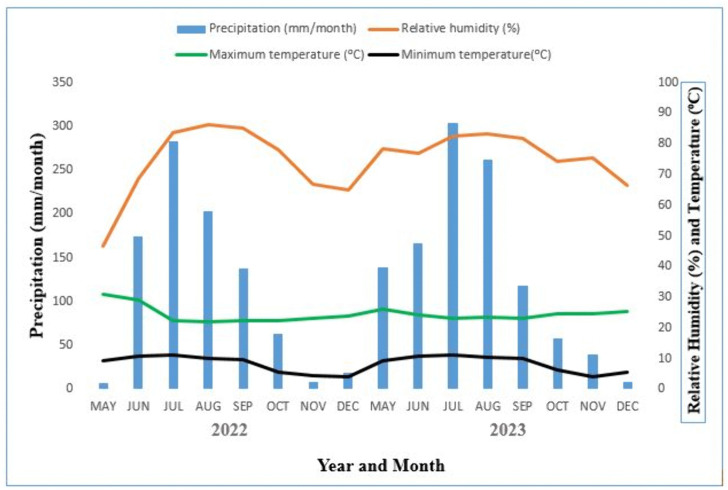
Weather data (2022 and 2023) of growing seasons at experimental site.

Two sets of field experiments were conducted during the 2022 and 2023 cropping seasons at Holetta Agricultural Research Center (HARC) in the Welmera district of the Oromia Regional State, Ethiopia. The experimental site is located about 30 km west of Addis Ababa at an altitude of 2400 meters above sea level. The experimental site (HARC) is located at 9° 3′ 19.43′′ N, 38° 30′ 25.43′′ E, and 30 km west of Addis Ababa, at an altitude of about 2400 meters above sea level (Fig 1). The area receives an average annual rainfall of1100 mm, with most precipitation occurring from June to September. The average minimum and maximum air temperatures are 6.2°C and 22.1°C, respectively [29]. The dominant soil type at the study area is Eutric Nitisols, and the district is one of the major barley-producing areas of the central Ethiopian highlands.

### Experimental materials and design

Two rows of the malt barley variety Ibon174/03 were used as test crops for this study. Compost was used as an organic fertilizer source in the experiment. Urea [CO (NH_2_)_2_] (46% N) and triple superphosphate (46% P_2_O_5_) were used as nutrient sources of nitrogen and phosphorus fertilizers, respectively. The experiment was a factorial randomized complete block design with the treatments studied being five N levels (0, 23, 46, 69, and 92 kg N ha^-1^) and four compost levels (0, 2.5, 5, and 7.5 t ha^-1^) and three replications for each treatment. A gross plot size of 2 x 3 m was used, and each treatment had 15 rows, each 3m long and 20 cm between rows. The net plot size was 2 x 2.6 m (central 11 rows of 3 m length) leaving the two outermost rows on both sides of each plot and 0.5 m row length at both ends of each plot to avoid border effects.

The spacing between plots and blocks was 0.5 m and 1 m, respectively. Triple superphosphate (46% P) was used as a source of phosphorus fertilizers. To avoid N losses by leaching, urea application was done in two splits, i.e., half at sowing time and the other half at the stages of tillering. The experimental fields were under potato and rapeseed cultivation in the years 2022 and 2023, respectively. The plots were kept free of weeds by hand weeding at different growth stages of the crop. Other agronomic practices were applied based on local research recommendations.

### Crop management

The experimental land was prepared by plowing. The 1^st^ ploughing was done by tractor, and the 2^nd^ and 3^rd^ ploughing by using oxen. Before sowing, the field was cleaned and prepared properly to receive treatments. Field layout was done based on the design of the experiment, and treatments were assigned to each experimental plot randomly. Seed was sown on 16 July 2022 and 8 July 2023 by hand drilling seeds in the rows at a recommended rate of 125 kg ha^-1^. Phosphorus as TSP (46% P_2_O_5_) was applied in the rows at the time of sowing, and nitrogen was applied based on the nature of treatments. Compost was applied to all plots (except the control) one month before planting and incorporated into the upper 15–20 cm of the soil layer. Furthermore, during both growing years, no disease and insect infestations were occurred in the experimental fields.

### Data collection and measurement

Thousand kernel weights was determined by taking three samples of thousand seeds weight taken from the grain yield of each net plot of experimental field. Seeds were counted using an electronic seed counter and weighed with a sensitive balance, and adjusted to 12.5% moisture content of the grain. Grain moisture content was measured by using apparatus Grain Analysis Computer (GAC) 2100) (perusing protein analysis computer) as described in the AACC (2000) method. Hectoliter weight was determined on dockage-free samples using a standard laboratory hectoliter weight apparatus (grain analysis computer (GAC)) as described in the AACC (2000) method. The protein content analysis was analyzed at the Holetta Agricultural Research Center laboratory using the standard operating procedures were measured by the Kjeldahl method (grain analysis computer (GAC) 2100) (perusing protein analysis computer) as described in the AACC (2000) method. Malt β-glucan was determined using the Mixed-Linkage β-Glucan Assay Kit according to the streamlined procedure**,** following AOAC Method 995.16 and AACC Method 32−23. Germination capacity was determined from 100 seeds germinated in a Petri dish after 72 hours. The germinated kernels are counted and the result is expressed as a percentage of the total. Germination energy was tested simultaneously with germination ability, and it was carried out on day four. The seeds that sprouted on day four after sowing were counted, and the proportion of sprouted seeds was calculated as a percentage.

### Statistical analysis

All collected data were subjected to the analysis of variance (ANOVA) procedure. Prior to analysis, normality of residuals was tested using the Shapiro–Wilk test, and homogeneity of variances was tested using Levene’s test residuals were tested. Once these assumptions were satisfied, the collected data was subjected to the ANOVA using R statistics software (ver. 3.4.1, 2017). A combined analysis of the two-year data was performed after testing the homogeneity of variances using an F-test, as described by [39].

### Ethics statement

This study did not involve human participants or animals. No specific permits were required for conducting the field experiments, and the research complied with institutional, national, and international guidelines for the responsible conduct of agricultural field studies.

## Results

### Thousand seed weight

**Fig 2:**
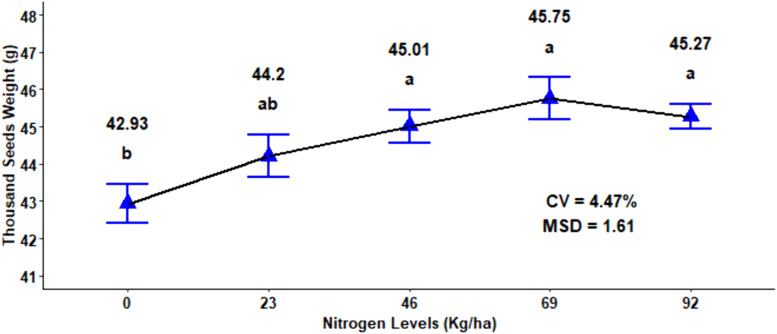
Main effects of N fertilizer rates on thousand seed weight.

The analysis of variance demonstrated that thousand seed weight (TSW) was very highly significantly affected by nitrogen fertilizer rate (P < 0.001) and by the two-way interaction of N rate and growing season. Besides, the two-way interaction of compost and growing season had a significant effect (P < 0.01) on the thousand seed weight of malt barley grain. However, the main effect of compost rates, the main effect of growing season, and the remaining two- and three way interaction had a significant effect on thousand seed weight (Table 1). The maximum TSW of (45.75 g) was recorded at N rates of both 92 kg ha ⁻ ¹ and 69 kg ha ⁻ ¹, and these values were statistically similar to the N rate of 46 kg ha^-1^ (Fig 2). Moreover, even a small application of 23 kg ha ⁻ ¹ N fertilizer increases in TSW from 42.93 g under zero or nil nitrogen to 44.20 g, indicating that even low nitrogen rate applications positively affect TSW (Fig 2).

**Table 1:** The effects of organic compost and nitrogen fertilization rates on selected quality of malting barley grown in the central highland of Ethiopia.

Factor	TSW	MC	HL	MAEX	GPC	Fria	Gluc	GC	GE
Nitrogen	29.37***	0.074^NS^	2.36^NS^	19.16***	10.02***	56.64***	102341***	52.40***	81.19***
Compost	7.74^NS^	0.052^NS^	12.99^NS^	13.90***	4.80***	19.78***	49044***	17.37**	7.89***
Year	10.27^NS^	2.55***	192.81***	90.48***	3.55***	0.87^NS^	42175***	0.09NS	3.24^NS^
N*Year	37.69***	0.131^NS^	14.32**	2.03***	0.09^NS^	0.20^NS^	278^NS^	2.49NS	1.01^NS^
N*Com	5.01^NS^	0.078^NS^	5.10^NS^	1.23**	0.11^NS^	0.91^NS^	801^NS^	11.67***	1.25^NS^
Com* Year	17.05**	0.0569^NS^	13.35^NS^	1.71**	0.35**	3.92*	2339*	2.76NS	7.85***
N*Com*Year	5.06^NS^	0.146*	5.25^NS^	0.48^NS^	0.17*	0.79^NS^	774^NS^	0.63NS	1.08^NS^
MSE	3.99	0.077	5.06	0.51	0.078	1.11	592	1.89	0.96
CV (%)	4.47	2.38	3.55	0.90	2.53	1.33	4.71	3.30	1.03

TSW = thousand seeds weight, MC = moisture content, HL = hectoliter weight, MAEX = malt extract yield, GPC = grain protein content, Fria = Malt friability, Gluc = beta-glucan, GC = germination capacity percentage, and GE = germination energy. Means within a column followed by the same letter are not significantly different at the 5% probability level *, **, *** and NS, significant, highly significant and non-significant, respectively.

**Fig 3:**
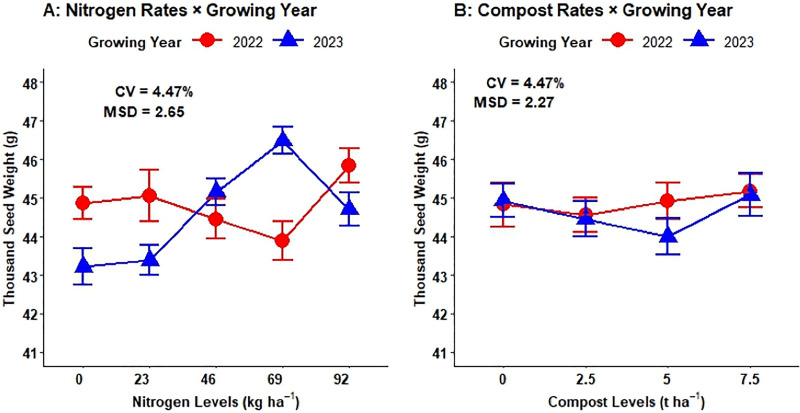
Interaction effects of N and compost rates by growing years on thousand seed weight.

The two-way interaction effects of mineral N and growing years highly affected (P < 0.001) the thousand seed weight. The highest (45.83 g) mean value of TSW was obtained at 92 kg ha^-1^ N fertilization rates during the 2022 growing season, whereas the lowest (41.48g) mean of thousand seed weight was obtained from plots that had no mineral N rates during the 2023 growing years (Fig 3A). The two-way interaction effects of compost rates and growing years were highly affected (P < 0.001) the thousand seed weight. The highest (45.88 g) mean value of TSW was obtained at 7.5t ha^-1^ a full dose of compost rates during the 2022 growing season, whereas the lowest (43.12 g) mean of thousand seed weight was obtained from plots that had was produced on plots treated with 5 t ha ⁻ ¹ during the 2022 cropping season (Fig 3B).

### Moisture content

**Fig 4:**
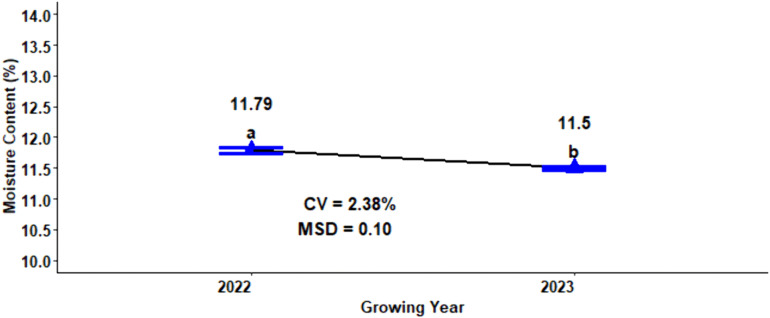
Effect of growing years on grain moisture content of malt barley.

The moisture content is one of the quality parameters that affect the quality of the product, storage, and processing of malt barley. The analysis of variance for moisture content of malt barley revealed that, excluding growing years, both the main and interaction effects of compost and nitrogen fertilizer rates over growing seasons were not significant (p < 0.05) (Table 1). On the other hand, the three-way interaction effect of N fertilizer rates, compost application rates, and growing seasons was statistically significant at (p < 0.05) on the moisture content of malt barley. The highest mean value of moisture content (11.79%) was recorded during the 2022 cropping years, while the smallest mean value of moisture content (11.50%) was obtained during the 2023 growing years (Fig 4).

**Fig 5:**
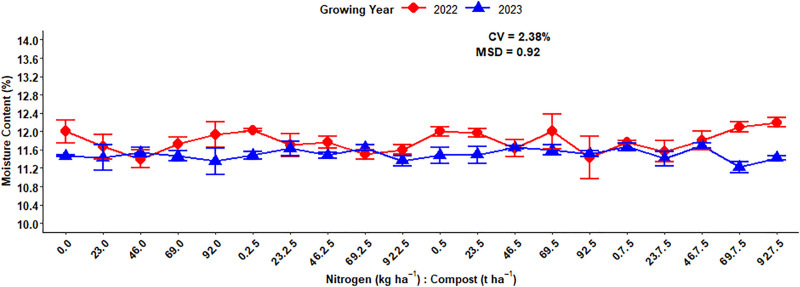
Three-way interaction effect of compost, N fertilizer rates, and growing year on moisture content.

The analysis of variance revealed that the interaction effect of organic (compost) and N fertilizer rates showed a significant difference in the moisture content of barley grain which means the influence of compost fertilizer rates depends on the application rate of N fertilizer. The maximum mean value of moisture content (12.20%) was recorded with 7.5 t ha^-1^ applied compost and 92 kg ha^-1^ nitrogen fertilizer rates during the 2022 cropping season, whereas the lowest mean of moisture content (11.22%) was obtained from plots that had the combination of 7.5 t ha^-1^ compost and 92 kg ha^-1^ nitrogen fertilizer rates during the 2023 growing season (Fig 5).

### Hectoliter weight

**Fig 6:**
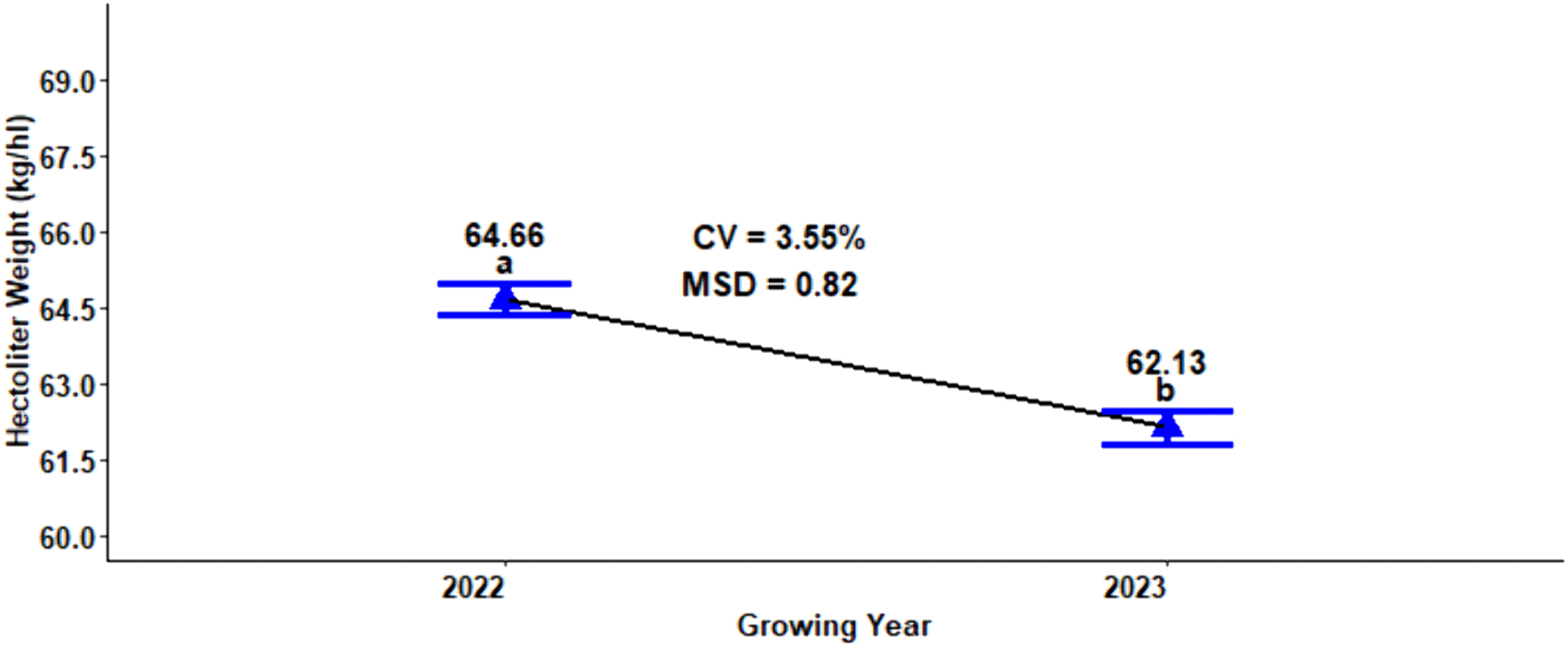
Effect of growing years on hectoliter weight of malt barley grain.

Analysis of variance revealed that hectoliter weight was very highly significant (P < 0.001), affected by the main effect of the growing season and significantly affected by the interaction effect of nitrogen and growing season (Table 1). The maximum (64.66 kg hl^-1^) and minimum (62.13 kg hl^-1^) hectoliter weights were scored in the 2022 growing season and 2023 growing season, respectively (Fig 6).

**Fig 7:**
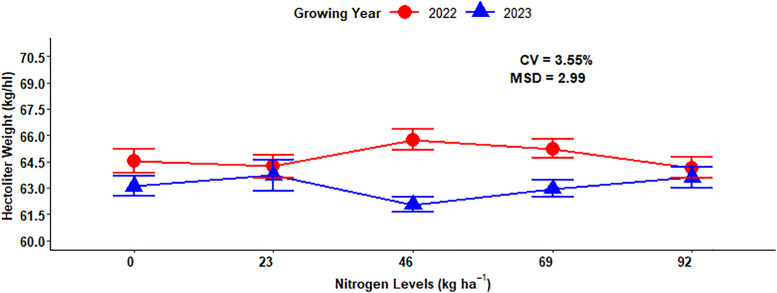
Two-way interaction effect of nitrogen rates and growing year on hectoliter weight.

The combined mean values of hectoliter weight of the growing season and rate of nitrogen fertilizer application show the highest hectoliter weight (65.75 kg hl^-1^) was recorded at 46 kg nitrogen fertilizer application during the 2022 growing season, while the lowest (61.03 kg hl^-1^) hectoliter weight was recorded at 46 kg ha^-1^ nitrogen fertilizer application during the 2023 growing season (Fig 7).

### Malt extract yield

The analysis of variance revealed that malt extract had a highly significant (P < 0.001) difference because of the main effects of nitrogen fertilizer, organic compost, and growing seasons. Besides, the malt extract was also significantly influenced by the interaction effects of nitrogen and compost fertilizer (P < 0.01), nitrogen and growing season (P < 0.05), and compost and growing season (P < 0.05) of malt barley. However, this parameter was not significantly affected by the interaction effects of fertilizer rates of nitrogen, compost, and growing season (Table 1).

**Fig 8:**
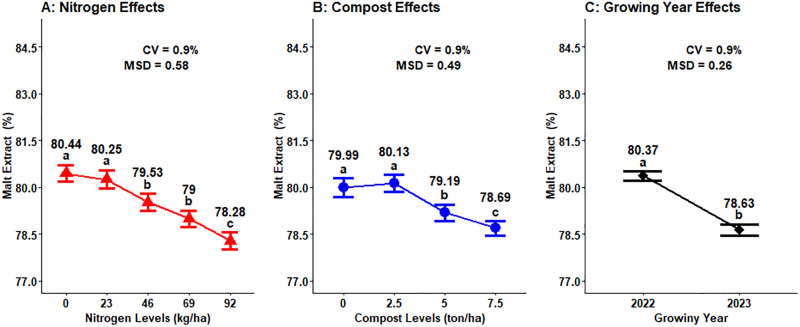
Main effect of nitrogen, compost rates and growing year on grain malt extract yield.

The highest malt extract (80.44%) of malt barley was measured from the lowest N fertilizer application (0 kg ha^- 1^), followed by a 23 kg ha^- 1^ nitrogen fertilizer application, which was 80.25%, while the lowest malt extract (78.28%) was observed from the highest nitrogen fertilizer rates (92 kg ha^- 1^), which was statistically different from all the other nitrogen fertilizer rates (Fig 8A). The highest malt extract (80.13%) of malt barley was observed from the lowest organic compost application rates (2.5 t ha^- 1^), followed by 0 t ha^- 1^ of compost rates, which was 79.99%, while the lowest malt extract (78.69%) was recorded from the highest compost fertilizer rate (7.5 t ha^- 1^), which was statistically different from all the other compost rates (Fig 8B). The malt extract of malt barley in the growing seasons was significantly different, with the highest malt extract (80.37%) recorded from the 2022 cropping season, while the lowest malt extract (78.63%) was observed in the 2023 growing season (Fig 8C).

**Fig 9:**
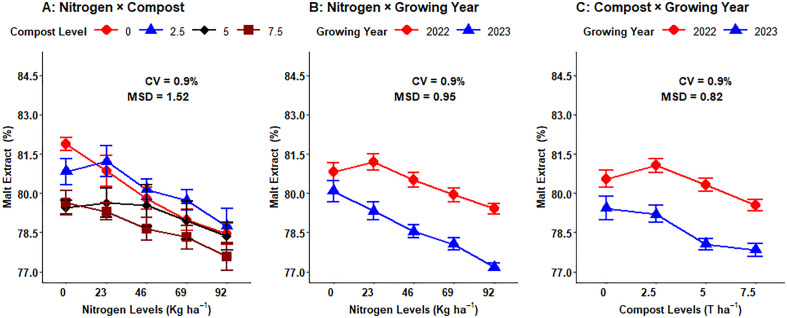
Two-way Interaction effects of N and compost rates, N rates and growing year, compost rates and growing season on grain malt extract.

The combined use of nitrogen and compost fertilizer rates significantly impacts the malt extract in malt barley grain. The malt extract was significantly influenced by the interaction effects of nitrogen and compost fertilizer (P < 0.01). The highest mean (81.87%) of malt extract was recorded from non-fertilized plots or control treatments, whereas the lowest mean (77.58%) of malt extract was observed from the combination of 92 kg N ha^-1^ with 7.5 t ha^-1^ (Fig 9A). The two-way interaction effects of nitrogen rates and growing years significantly affect the malt extract in malt barley grain. The highest mean (81.19%) of malt extract was recorded from 23 kg N ha^-1^ during the 2022 growing year treatments, whereas the lowest mean (77.16) of malt extract was observed from the application of 92 kg N ha^-1^ during the2023 growing year (Fig 9B). The two-way interaction effects of compost rates and growing years significantly affect the malt extract in malt barley grain. The highest mean (81.06%) of malt extract was recorded from 2.5 t ha^-1^ of compost rates during the 2022 growing year treatments, whereas the lowest mean (77.84%) of malt extract was recorded from the application of 7.5 t ha^-1^ of compost amendments during the 2023 growing years (Fig 9C).

### Grain protein content

**Fig 10:**
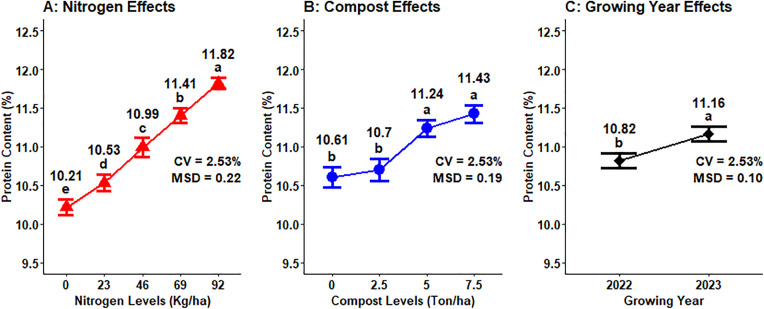
Main effect of nitrogen, compost rates and growing year on grain protein content.

The grain protein concentration of malt barley grain was highly significantly (P < 0.001) influenced by the sole effect of mineral N, compost rates, and growing years. Besides, the interaction effect of organic compost, nitrogen rates, and growing seasons significantly (P < 0.05) impacted the protein content of malt (Table 1). The grain protein concentration levels are positively correlated with N fertilizer rates, which is increased protein content of the grain with N fertilizer rates. The maximum (11.82%) grain protein concentration was measured from the maximum (92 kg N ha^-1^) application of N fertilizer rates, whereas the lowest protein level was recorded from non-fertilized plots (Fig 10A). Plots fertilized with a maximum (7.5 t ha^-1^) compost rate gave the highest (11.43%) protein level of malt barley grain, whereas unfertilized plots with compost resulted in the lowest (10.61%) grain protein concentration, which shows that an increase of compost fertilizer levels raises the grain protein concentration of malt barley in the study area (Fig 10B). The highest (11.16%) grain protein content was obtained during the 2023 main cropping season, whereas the lowest (10.82%) grain protein concentration was recorded during the main cropping season (Fig 10C).

**Fig 11:**
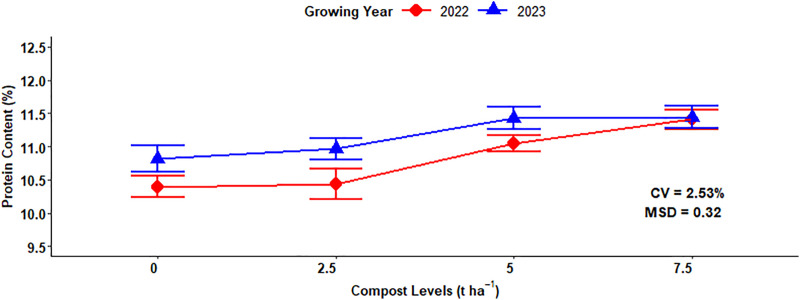
Two-way interaction effect of compost rates and growing years on grain protein content.

The maximum malt grain protein concentration (11.44%) in the 2023 growing year was recorded from 7.5 t ha^-1^ of compost application, while the lowest (10.40%) malt grain protein content in the 2022 growing season was obtained from 0 t ha^-1^ of compost application, which was statistically similar to the grain protein accumulation recorded in 2022 at 2.50 t ha^-1^ of compost application rate (Fig 11).

**Fig 12:**
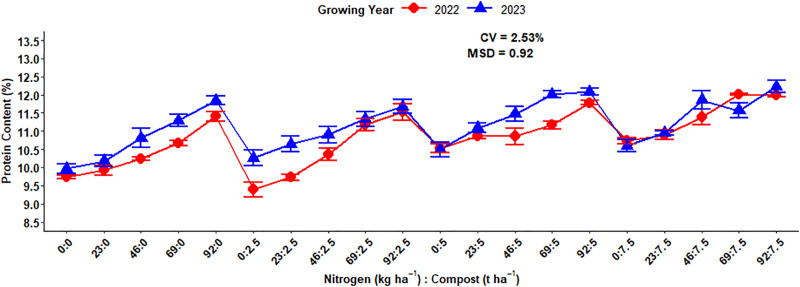
Three-way interaction effect of compost, N fertilizer rates, and growing year on grain protein content.

The highest (12.33%) grain protein concentration was recorded at the highest input application of 92 kg N ha^-1^ combined with 7.5 t ha^-1^ compost rates during the 2023 growing years while the lowest (9.39%) grain protein content was recorded at nil application of N fertilizer with 2.5 t ha^-1^ of compost rates during the 2022 growing year (Fig 12).

### Malt friability content

**Fig 13:**
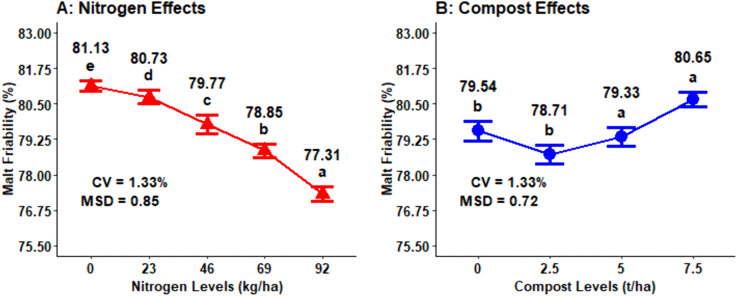
Main effect of nitrogen and compost rates on malt friability.

The ANOVA analysis revealed that the main effect of chemical N fertilization and compost fertilizer sources had a highly significant (P < 0.001) effect on the malt friability content of malt barley grain; however, the interaction effects were not significant except for the combination of cropping season and organic compost fertilizer levels (Table 1). The N fertilization levels are inversely correlated with the malt friability content and the highest obtained from the non-fertilized plots with N fertilizer rates (81.13%), followed by 23 kg N ha^-1^ (80.73%) and 46 kg N ha^-1^ (79.77%), whereas the lowest (77.81%) friability concentration was measured from the highest (92 kg N ha^-1^) chemical nitrogen fertilizer rate (Fig 13A). The highest (80.65%) mean malt friability was recorded from the application of 7.5 t ha^−1^ of compost rate, while the lowest (80.65%) malt friability was obtained from the application of 2.5 t ha^−1^ of compost rate (Fig 13B).

**Fig 14:**
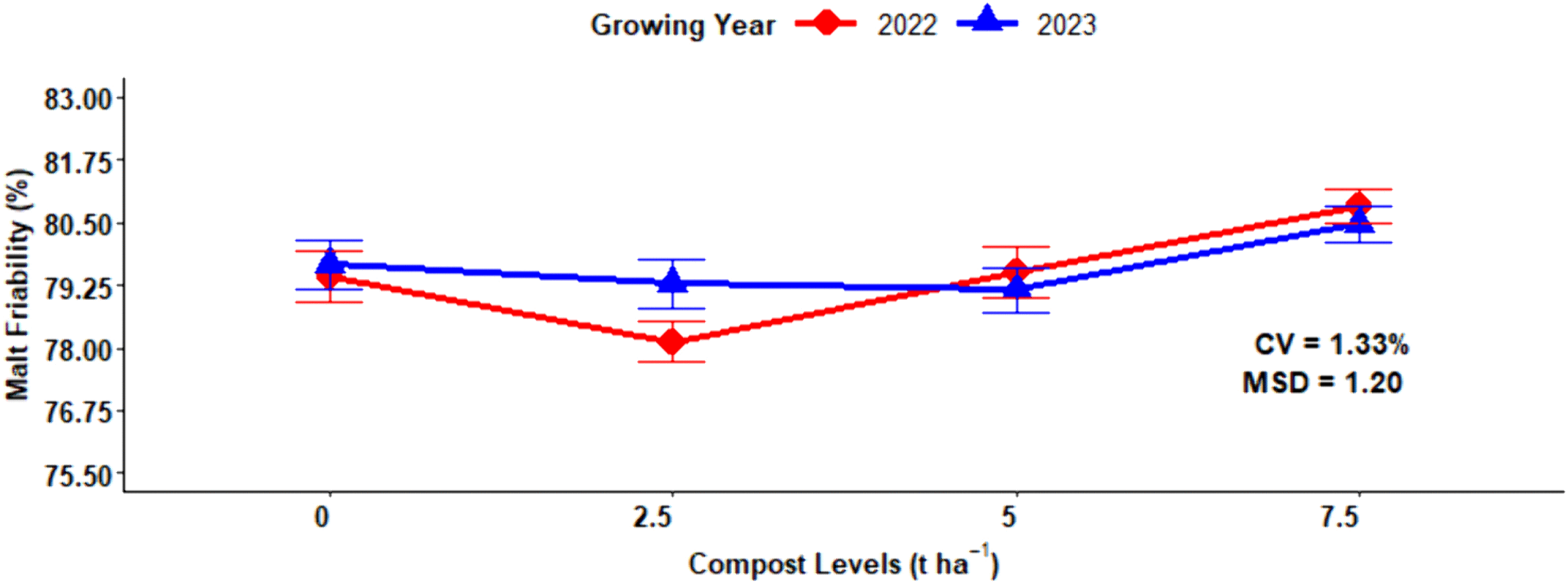
Two-way interaction effect of compost rates and growing years on malt friability.

The maximum malt friability value (80.84%) was observed in the 2022 growing years at 7.5 t ha^-1^ of compost application rate, while the minimum (78.12%) was obtained from 2.5 t ha ⁻ ¹ of compost applied in the 2022 growing years. In the 2023 growing year, the maximum value (80.47%) of malt friability was also observed at 7.5 t ha ⁻ ¹, showing that maximum compost rates consistently improved malt friability across both growing years (Fig 14).

### Malt β-glucan

**Fig 15:**
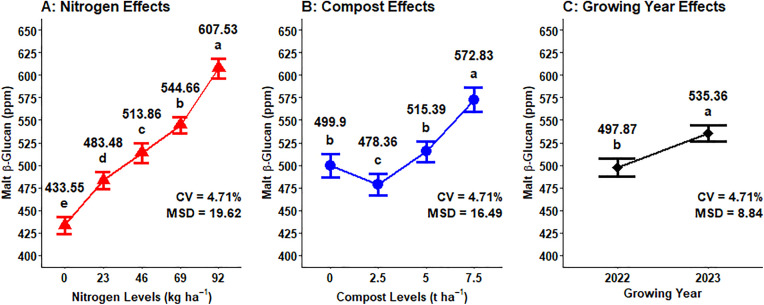
Main effect of nitrogen, compost rates and growing year on grain malt β-glucan.

The analysis of variance showed that the beta-glucan of malt barley was highly significant (P < 0.001) influenced by the main effect of mineral N, organic compost, and growing year. Additionally, the interaction of growing year and compost fertilizer levels significantly (P < 0.05) affected the malt beta-glucan of malting barley grain. However, other interaction effects did not significantly impact the β-glucan of malt barley (Table 1). The maximum (607.53 ppm) malt β-glucan was obtained from the plots fertilized with 92 kg N ha^-1^ which was followed by (544.66 ppm) recorded from 69 kg N ha^-1^, whereas the lowest (433.55 ppm) malt β-glucan was observed from control or unfertilized plots (0 kg N ha^-1^) (Fig 15A). Regarding the main effect of compost fertilizer rate on malt β-glucan concentration, the highest (572.53 ppm) malt β-glucan content was obtained with the maximum application of compost (7.5 t ha^-1^), whereas the lowest (478.36 ppm) malt β-glucan was observed at the (2.5 t ha^-1^) compost application rate (Fig 15B). Regarding the effect growing year on malt β-glucan, the highest (535.36 ppm) was recorded in the growing year, while the lowest mean (497.87 ppm) of the malt β-glucan was obtained at 2022 growing year (Fig 15C).

**Fig 16:**
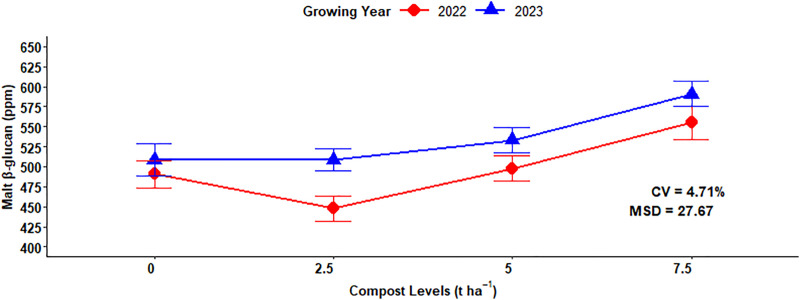
Two-way interaction effects of compost rates and growing years on grain malt β glucan.

Malt β-glucan content was significantly affected by compost fertilizer rate, with influences varying between growing years. The maximum malt β-glucan content (590.72 ppm) in the second year (2023) was recorded from 7.5 ton ha^-1^of compost application, and the lowest (447.89 ppm) malt β-glucan content in the first year of the growing season (2022) was obtained from 2.5 t ha^-1^ of the compost application (Fig 16).

### Germination capacity

The analysis of variance results indicated that the main effects of N fertilization and compost rates highly (P < 0.001) and significantly (P < 0.01) affected the germination capacity (GC) of malt barley kernels, respectively. Additionally, the interaction effect of mineral N and compost levels highly (P < 0.001) influenced the germination capacity. However, the other main and interaction effects did not significantly affect the germination capacity of malt barley (Table 1).

**Fig 17:**
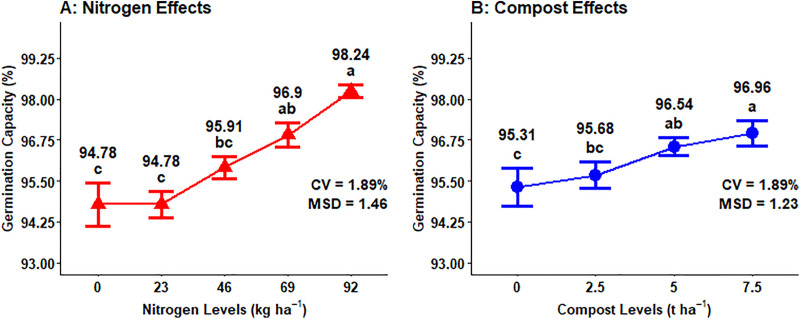
Main effect of nitrogen and compost rates on germination capacity.

The maximum (98.24%) germination capacity was recorded from the highest 92 kg N ha^-1^ mineral N fertilization rate, which was followed by 69 kg N ha^-1^ (96.90%), while the lowest (94.78%) germination capacity was from the control or unfertilized treatment, which was statistically similar to 23 kg N ha^-1^ (Fig 17A). The highest (96.96%) grain germination capacity was recorded from the highest 7.5 ton ha^-1^ application of compost level, while the lowest (95.31%) GC of barley grain was measured from unfertilized plots by compost (Fig 17B).

**Fig 18:**
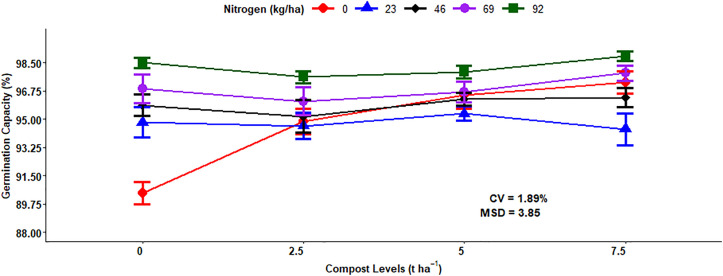
Two-way interaction effects of mineral nitrogen and compost rates on germination capacity.

The maximum (98.92%) germination capacity mean was recorded from the combined application of 92 kg ha^-1^ of N fertilization and 7.5 t ha^-1^ of compost fertilizer rates. However, the lowest (90.44%) germination capacity of malting barley grain was obtained from a control or unfertilized plot (Fig 18).

### Germination energy

**Fig 19:**
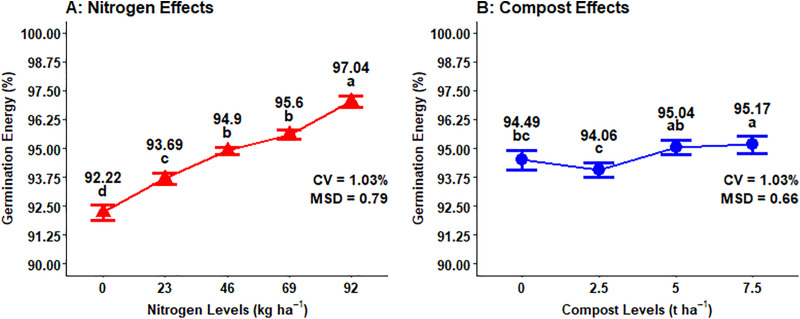
Main effects of mineral nitrogen and compost rates on germination energy.

The germination energy of malt barley grain was highly (P < 0.001) responsive to the main effect of N fertilization, compost rate, and interaction effects of compost fertilizer rate and growing year, while the main effect of growing year and other interaction effects did not significantly affect the germination energy (GE) of the malt barley grain (Table 1). The highest (97.05%) germination energy was recorded from 92 kg ha^-1^ of N fertilization levels and followed (95.60%) by 69 kg ha^-1^ chemical N fertilizer rate. While the lowest (92.22%) germination energy was obtained from the control or unfertilized plot (Fig 19A). The current finding showed that the GE of the malted barley fell within an acceptable range for both 69 kg ha^-1^ and 92 kg ha^-1^ of N fertilization levels. The main effect of compost application rates highly affected the germination energy of malt barley grain. The highest (95.17%) of GE of barley grain was recorded from the highest (7.5t ha^-1^) compost rate, while the lowest (94.49%) germination energy was obtained from unfertilized plots or treatments (Fig 19B).

**Fig 20:**
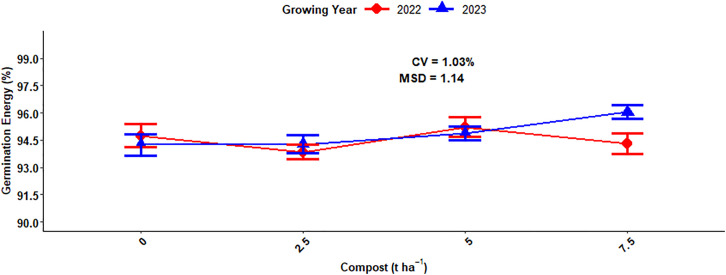
Two-way interaction effect of compost rates and growing years on germination energy.

The result of the analysis of variance showed that the combination effect of compost fertilizer rates and growing year highly significantly affected the germination energy of malt barley grain. Specifically, the highest (96.04%) germination energy of malting barley was recorded in the 2023 growing season with the application of (7.5 t ha^-1^) compost fertilizer rates and also closely followed by (5 t ha^-1^) compost rates in the 2022 growing season. However, the lowest (93.84%) germination energy was obtained in the 2022 growing season with a compost application rate of (2.5 t ha^-1^) (Fig 20).

### Multiple regression analysis of malt barley quality parameters

#### Multiple regression analysis for grain protein concentration.

**Fig 21:**
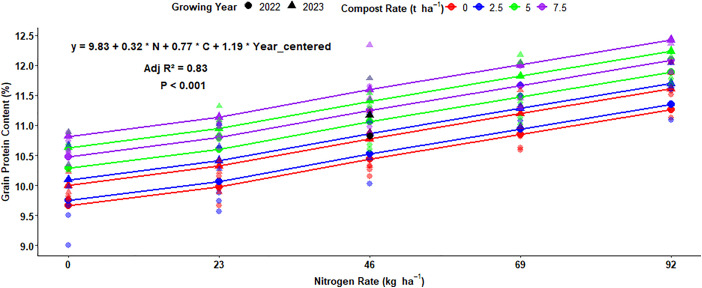
Multiple linear regression of grain protein content as influenced by nitrogen, compost, and growing year.

The analysis of multiple linear regression showed a positive effect on the grain protein content of malt barley. There was an expected average protein concentration of 9.73% for malt barley grain under field conditions with no inputs and no change in growing years. An increase of N rate by 1 kg ha^-1^ will raise the protein concentration by 0.02% (Fig 21). There was a stronger effect of compost rate than N fertilization rate, which found that an application of each t ha^-1^ of organic compost raised the grain protein content by 0.12% (Fig 21). Moreover, the change in the growing year also has a significant effect on the protein concentration of malt barley grain and raises protein content by 0.34% (Fig 21).

### Multiple regression for malt extract yield

**Fig 22:**
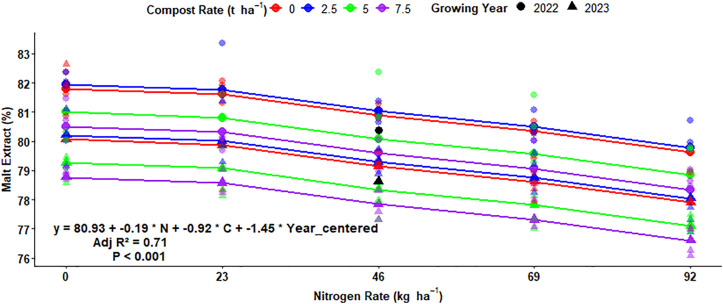
Multiple linear regression of malt extract yield as influenced by nitrogen, compost, and growing year.

The regression analysis equation showed that the malt extract levels decrease in boosting N fertilization, organic rates, and change in growing years. The difference in malt extract of the grain affected about 71%, which was indicated by adjusted r-squared (0.69) (Fig 22). There was an expected average malt extract yield of 80.93% for malt barley grain under field conditions with no inputs and change in growing years. The findings show that increasing of N rate by 1 kg ha^-1^ reduces the malt extract yield by 0.19%, holding other factors constant (Fig 22). In addition, increasing the compost rate by 1 t ha^-1^ reduces the malt extract yield by 0.92% (Fig 22). Moreover, the change in the growing year also has a significant effect on the malt extract yield of malt barley grain and reduces malt extract by 1.45% (Fig 22).

### Multiple regression for malt friability

**Fig 23:**
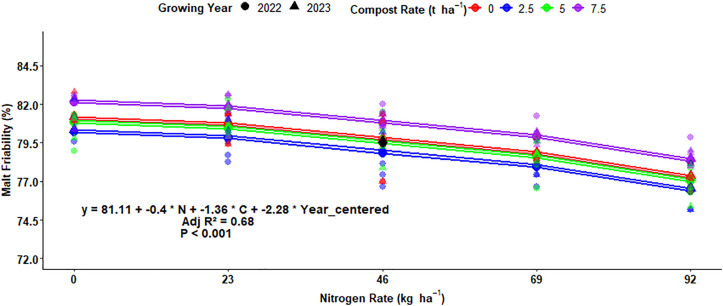
Multiple linear regression of malt friability as influenced by nitrogen, compost, and growing year.

The regression analysis equation demonstrated that the malt friability percentage was affected by changing N fertilization, organic rates, and growing years. The difference in malt extract of the grain affected about 68%, which was indicated by adjusted r-squared (0.68) (Fig 23). Rising the N fertilization rate has a negative correlation with malt friability, which shows that elevating N fertilization rates decreases malt friability percentage. The results show that increasing the N rate by 1 kg ha^-1^ reduces the malt friability by 0.4%, holding other factors constant. Regarding compost rates, increasing the compost rate by 1 t ha-1 reduce the malt friability by 1.36% (Fig 23). Moreover, the change in the growing year also has a significant effect on the malt friability of malt barley grain and reduces malt friability by 2.28% (Fig 23).

### Multiple regression for beta-glucan of malt barley grain

**Fig 24:**
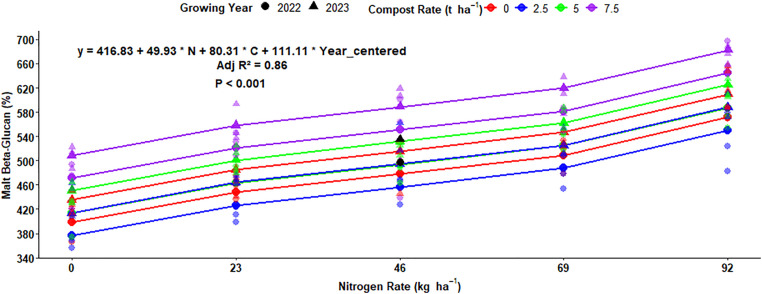
Multiple linear regression of malt beta glucan as influenced by nitrogen, compost, and growing year.

The multiple regression analysis equation showed that the beta-glucan concentration in malt barley was highly affected by nitrogen fertilization, organic compost rates, and growing years. The difference in beta-glucan content of the grain affected about 86% of the variation, which was explained by these three factors (Fig 24). The regression analysis equation showed that for every 1 kg ha^-1^ rise in N fertilization, beta-glucan content increases by 49.93 ppm (Fig 24). Regarding compost rates, increasing of compost rate by 1 t ha^-1^ increase the malt beta glucan by 80.31 ppm (Fig 24). Moreover, the change in the growing year also has a significant effect on the malt beta glucan of malt barley grain and increases malt beta glucan by 111.11 ppm, holding other factors constant (Fig 24).

### Multiple regression for germination energy of malt barley grain

**Fig 25:**
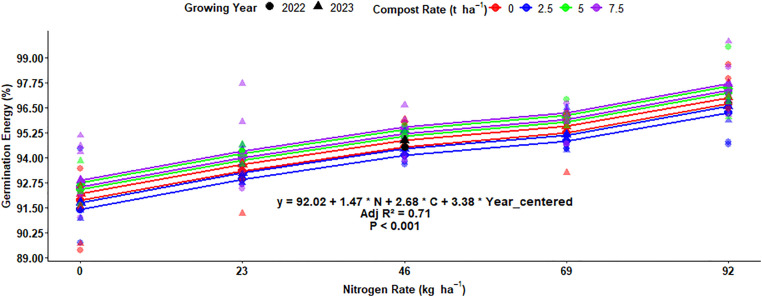
Multiple linear regression of germination energy as influenced by nitrogen, compost, and growing year.

The multiple regression analysis equation showed that the germination energy in malt barley was highly affected by nitrogen fertilization, organic compost rates, and growing years (Fig 25). The difference in germination energy of the grain affected about 71% of the variation, which explained by these three factors (Fig 25). The regression analysis equation showed that for every 1 kg ha^-1^ rise in N fertilization, germination energy increases by 1.47% (Fig 25). Similarly, each additional ton ha^-1^ of organic compost results in a 2.68% increase in the germination energy of malt barley (Fig 25). Moreover, the influence of growing years has a considerable influence, with an increase of 3.38% of germination energy per growing year showing that environmental conditions across years play a significant role (Fig 25).

### Correlation analysis of quality parameters on malt barley

Protein concentration of malt barley grain was positively and highly significantly correlated with beta-glucan (r = 0.83***), germination energy (r = 0.71***), and germination capacity (r = 0.55***); but malt extract yield (r = −0.71***) and malt friability (r = −0.43***) were negatively and highly significantly correlated with protein content. Germination energy exhibited a positive and highly significant correlated with beta-glucan (r = 0.75***) and germination capacity (r = 0.49***); however, malt extract yield (r = −0.58***) and malt friability (r = −0.54***) were negatively and highly significantly correlated with germination energy (Table 2). Malt extract yield exhibited a negative and highly significant correlation with beta-glucan (r = −0.71***) and germination capacity (r = −0.44***); however, malt friability (r = 0.24**), moisture content (r = 0.24**), and hectoliter weight (r = 0.29**) were positively and significantly correlated with malt extract yield. Beta-glucan of malt barley grain was positively and highly significantly correlated with germination capacity (r = 0.52***) and significantly correlated with thousand-seed weight (r = 0.27**); but malt friability (r = −0.38***) was negatively and highly significantly correlated with beta-glucan (Table 2).

**Table 2:** Correlation among grain quality parameters of malt barley.

Parameters	TSW	MC	HL	MaEX	GPC	Fria	Gluc	GC	GE
TSW	1								
MC	0.17^NS^	1							
HL	0.21**	0.24**	1						
MaEX	−0.12 ^NS^	0.24**	0.29**	1					
PC	0.31***	−0.1^NS^	−0.17^NS^	−0.72***	1				
Fria	−0.13^NS^	0.06^NS^	−0.11^NS^	0.24**	−0.43***	1			
Gluc	0.27**	−0.14^NS^	−0.16^NS^	−0.71 ***	0.83***	−0.38***	1		
GC	0.11^NS^	−0.09^NS^	0.015^NS^	−0.44 ***	0.55***	−0.39***	0.52***	1	
GE	0.27**	−0.03^NS^	−0.052^NS^	−0.58***	0.71***	−0.54***	0.75***	0.49***	1

*, **, *** Significant at p ≤ 0.05, 0.01, and 0.001 probability levels: NS: Not significant. TSW = Thousand Seeds Weight, MC = Moisture Content, HL = Hectoliter Weight, MaEX = Malt Extract, GPC = Grain Protein Content, Fria = Malt Friability, Gluc = Beta-glucan, GC = Germination Capacity, GE = Germination Energy.

## Discussion

### Thousand seeds weight

The results indicated that as the N fertilizer rate increases, the thousand-seed weight also tends to increase, with notable statistical significance among the different treatment levels. The positive response of thousand seed weight to increasing N rates shows the importance of nitrogen in improving barley seed development. This might be related to N fertilizer at a higher rate promoting better nutrient uptake (increases nutrient availability) and enhancing photosynthesis (promotes chlorophyll production) and robust vegetative growth (greater leaf area and more developed root systems), resulting in improved seed development and quality of seed in malt barley. The current finding is highly agreed with result of Kassie and Tesfaye [40] revealed that the thousand seed weight of malting barley grain increased as synthetic N fertilizer increased in the Arsi Highlands of Ethiopia. Similarly, Azam *et al.* [41], who stated that the application of synthetic nitrogen fertilizer of 120 kg N kg^-1^ increases the thousand kernel wheat of bread wheat during two growing seasons. The highest thousand grain weight was recorded at the highest application of mineral nitrogen rate on durum wheat under Algerian semi-arid conditions [42].

The maximum thousand-kernel weight obtained, probably due to the response to N fertilizer, can differ significantly between cropping seasons, most likely due to variations in weather conditions such as temperature, rainfall, and soil moisture, which can influence nutrient uptake and plant growth. This result was supported by Cammarano *et al.* [17] who revealed that the growing years or cropping seasons affected the thousand-seed weight of malting barley at the James Hutton Institute.

The optimum dose of organic amendment positively affects thousand seed weight by improving soil health, nutrient accessibility, and soil physical properties together with favorable weather conditions for plant growth. This result was agreed with Omirou *et al.* [43], who reported that compost can provide essential nutrients needed for plant growth and development. The difference may result from seasonal variations in weather conditions, such as rainfall and temperature, which can influence nutrient availability and finally affect plant growth and seed weight of the crop. These results corroborate the previous finding of Omirou *et al.* [43], who suggested that the kind of N fertilizer source influences the accumulation of essential plant nutrients and its effect varies depending on the growing season.

### Moisture content

The differences in weather conditions (temperature and rainfall), and cooler temperature or reliable rainfall during the cropping season can improve soil moisture retention and decrease evaporation, which is leading to higher moisture content of the final grain; however, the cropping season with higher temperature or lower rainfall might have a lower moisture content of the grain than normal conditions. This aligns with the consistent finding of Bahmani *et al.* [44], who stated that weather conditions are the main factors that can hinder malt barley yield and malting characteristics. Likewise, Assefa *et al.* [13], suggested that the growth, yield, and yield parameters of food barley were significantly affected by the growing season, most likely due to optimum rain distribution and a favorable temperature.

### Hectoliter weight

The difference in hectoliter weight of malt barley was due to growing condition variation during the growing season or cropping season. This is due to the fact that under more conducive cropping situations, there was the slight enhancement of specific hectoliter weight in response to nitrogen fertilizer application levels, and the small values of hectoliter weight show that there is poor growth of grain filling. In addition, the absolute plant yields and grain quality may differ from year to year due to the changeable weather situations of the specific area. Variations in weather conditions between growing seasons can significantly influence the grain quality of malt barley, including hectoliter weight. This result is in line with the study of Kassie and Tesfaye [40], who reported that seasonal condition significantly influenced hectoliter weight of malting barley grain. The present results indicated that all the nitrogen fertilizer rates with both the 2022 and 2023 growing seasons don’t achieve the acceptable hectoliter weight of the brewery requirement.

### Malt extract yield

Malt extract is the soluble substance formed by malt itself and enzymatic hydrolysis during saccharification and fermentation, which indicates the degree of malt dissolution and volume of enzyme production in the malting process [23]. Malt extract content is a key quality indicator because it reflects the amount of beer that can be produced from a given quantity of malt. Increasing nitrogen fertilizer rate application caused grain protein concentrations to rise, which limited endosperm modification and diminished malt extract concentration [45]. Besides, higher malt extract content is normally related to the low protein content of the crop. The control treatment of not applying any fertilizers, organic manure, or bio-fertilizers showed maximum content of malt crops [25]. On the other hand, Cammarano *et al.* [17] who demonstrated that the malt extract of malt barley grain did not indicate any noticeable variation due to change of cropping seasons.

Increasing nutrient content in the soil will diminish the malt extract content, which is negatively correlated to the grain protein content of malt barley. In addition, an increase in nutrient content in the soil may create undesirable qualities in the grain of malt barley, which is increasing levels of nitrogen fertilizer and organic compost rates may decrease the malt extract of the grain and increase the grain protein contents. This result was agreed with Reussi Calvo *et al.* [20], who observed that an application of nitrogen fertilizer nutrient decreased the malt extract content of malt barley by 0.8%.

### Grain protein content

The difference in grain protein accumulation is might be due to improved nutrient accessibility and enhanced soil condition because of higher compost application rates specifically in the 2023 growing years, which may have had conducive environmental conditions. The result of this experiment agreed with Shilev *et al.* [46], who revealed that sole application of vermicompost resulted the maximum protein concentrations, followed by integrated treatments and inorganic fertilizer.

Increasing mineral N fertilizer rates linearly increased the grain protein concentration of malt barley in the Arsi highlands of Ethiopia [40]. Similarly, Goettl *et al.* [21] revealed that the applications of nitrogen fertilizer levels have a strong positive relationship with grain protein content in North Dakota of two-row malting barley. Moreover, the highest rate of nitrogen fertilizer results in the highest concentration of grain protein of malt barley, as reported by different scholars [22,47,48]. Furthermore Habiyaremye *et al.* [49], who demonstrated that the nitrogen application had a significant influence on grain protein concentration in food barley, with protein contents rising positively in response to higher levels of mineral N fertilizer in the Palouse Region of the Pacific Northwest.

The mixed use of chemical N fertilizer along with organic amendments maintains or slightly decreases grain protein concentration as compared to full application of chemical N fertilizer [50]. Similarly, application of biofertilizers (Microbein + 75% NPK) resulted the maximum values of protein content of barley crop [51]. The standards set for a grain protein concentration of malt barley by the Ethiopian Standard Authority and Asella Malt Factory should ranges from 9–12% [52]. Therefore, the above range almost all fulfilled brewery requirements or acceptable range for beer factories. Growing year highly significantly affected the concentration of protein in malt barley grain due to high temperature during grain filling, especially during cell division in starch endosperm or increased starch accumulation, which resulted in maximum grain protein content [53].

### Malt friability content

The difference in malt friability values might be due to improved nutrient accessibility and enhanced soil microbial activity and better soil physical characteristics under maximum compost application rates. The relatively maximum higher malt friability values were recorded in the 2023 as compared to the 2022 growing years due to more conducive environmental conditions in the 2022 growing year which likely improved organic matter decomposition. The increased malt friability of malt barley kernels with decreased nitrogen fertilization rates. Malt friability of barley is highly affected by the rate of nitrogen fertilization, and an increase of N fertilizer rate decreased the malt friability of the grain. The current finding is in line with result of Cammarano *et al.* [17], who reported that the nitrogen fertilization rates highly affected malt friability and the treatment with maximum protein concentration of the grain reduces malt friability of malt barley. Similarly, malt friability was influenced significantly by diverse sources of nitrogen fertilizer [25].

### Malt β-glucan

Malt β-glucan of malting barley responded linearly, whereby increased rates of N fertilization resulted in boosting the malt β-glucan content. Rising nitrogen fertilization levels increase the malt β-glucan content of barley. A similar result was also reported by Habiyaremye *et al.* [49], who demonstrated that the malt β-glucan content was highly responsive to the interaction between mineral N rates along with food barley variety across all cropping seasons in Almota and Genesee experimental sites. However, combined application of organic fertilization with mineral N fertilizer rates had no significant influence on concentration of beta-glucan in barley and wheat crops [54].

Malt β-glucan concentration was significantly affected due to growing years, due to environmental conditions or weather variables, and soil moisture during grain development affects the role of enzymes involved in β-glucan synthesis and degradation. This result is in line with the finding of Khaleghdoust *et al.* [54], who revealed that malt β-glucan concentration was highly influenced by the weather condition of the cropping season, with seasonal variations causing large differences in the malt β-glucan of barley and wheat grain.

### Germination capacity

The percentage of viable grain is determined by germination capacity, which is one of the key features of malt barley quality because it determines the suitability for high-quality malt and beer production. The germination capacity of malt barley was highly affected by chemical N fertilizer application levels. The chemical N fertilization doses affected the germination percentage of barley seeds [26]. Similarly, Kristó *et al.* [55], who found that the application of mineral NPK rates significantly affected the seed quality of wheat grain including germination percentage and power. Regarding organic fertilizer sources, they are very important in improving the germination capacity of malt barley grain based on the dosage. Moreover, seed germination is highly influenced due to nitrogen addition [56].

### Germination energy

Germination energy was highly responded to the main effect of the chemical N fertilizer rate. This is due to the N providing essential nutrients that enhance embryo development and vigor, enhancing the seed metabolic process during germination. This result is inconsistent with finding of Nure [57], who reported that the application of chemical N fertilizer rate significantly boosted the germination energy of malt barley grain, with higher rates resulting in the higher germination energy. The GE of malting barley grain is recommended to be greater than 95% to have quality output [58].

#### Multiple regression analysis for grain protein concentration.

Regarding the effects of organic compost amendment rates, the protein concentration of malt barley grain showed significant responses for each rate. Regarding the effect of growing years on grain protein content, different scholars discovered similar results to the current study. The N fertilization rate and growing years highly affected the concentration of malt barley grain [16]. Increasing nitrogen accessibility due to elevating the N fertilizer level reduced grain protein content from 0 to 40 kg ha^-1^; however further N fertilizer application steadily increased it [16].

Moreover, Halstead *et al.* [48] the N-fertilization rates highly influenced the kernel protein accumulations in the Pacific Northwest (USA). Moreover, Tadesse *et al.* [19] discovered that N fertilization rates significantly affected the grain protein accumulation of malting barley, with a linear grain protein response at one of their experimental sites. The result of this experiment was in contrast with Mills *et al.* [59], who discovered that boosting the N fertilization rates from 0 to 120 kg ha^-1^ resulted in a reduction of malt protein concentration. The application of vermicompost alone results in a higher protein accumulation of barley grain [46].

Sainju [16] revealed that the growing years or seasons influenced the grain protein concentration, and the variation in kernel protein accumulations was due to favorable weather variables (higher precipitation) between growing seasons. In contrast to the current investigation, Kassie and Tesfaye [18] discovered that the growing season had no impact on grain protein concentration, showing that the experimental season didn’t present situations influencing grain protein accumulation of malting barley in the Arsi highlands of Ethiopia.

### Multiple regression for malt extract yield

Declines in malt extract levels were noted when N fertilization application was elevated which was similar to Rogers *et al.* [24], who reported that boosted N fertilization application rates led to small falls in malt extract of malt barley grain. However, Adeyemi [60], demonstrated that elevating the N fertilizer rates boosts the malt extract of malt barley grain and also showed that the treatment with 180 kg ha^-1^ had 1–2% higher than the control or check plot at Aberdeen in the 2022 growing year.

The change in experimental seasons affected the malt extract of malt barley grain and malt extract in the 2022 growing year much more than in the 2023 growing year. This might be due to changes in weather conditions (fluctuations in temperature and patterns of rainfall) during growing years. These weather alterations directly affected the kernel compositions by changing protein-to-starch ratios, which were particularly determined by malt extract of barley grain. Boosted protein concentrations under sub-optimal conditions reduce starch accumulation, thus reducing hot water extract (malt extract) yield. The malt extract of different malt barley cultivars was affected by growing years in 2015/16 and 2016/17 in Turkey under rainfed agriculture [61].

### Multiple regression for malt friability

The improved soil physical and biological properties through compost application enhance the malt friability of malt barley grain. Boosting N levels reduces the malt friability of malt barley grain [62]. However, Parashar *et al.* [63] discovered that application of 120 and 90 kg N/ha N fertilization rates boosted the average malt friability by 13.8% and 12.6% over 60 kg N ha^-1^, respectively. The malt friability percentage of the spring barley was highly varied due to experimental growing years [17].

#### Multiple regression for Beta-glucan of malt barley grain.

The wort beta-glucan of barley was influenced by N fertilization rates, and boosting N rates from 0−120 kg ha^-1^ elevated the wort beta-glucan concentration from 77.6–208.1 mg L^-1^, respectively [59]. However, Habiyaremye *et al.* [49] reported that application of N fertilization had no significant impact on beta glucan across different growing years as well as experimental sites. Regarding the impact of the growing season on beta-glucan of the present result, a similar investigation was discovered by Khaleghdoust *et al.* [54] who showed that beta-glucan concentration of malt barley highly responded to the growing year’s conditions. Similarly, Kumar *et al.* [53] discovered that the beta-glucan was highly influenced by the main effect of experimental seasons, such that each the growing year was characterized by a specific set of weather variables like temperature, humidity, rainfall, and sunlight.

### Multiple regression for germination energy of malt barley grain

Germination energy of malt barley significantly responded to the main effects of nitrogen fertilization, compost, and growing years. Nitrogen fertilizer levels had significant effect on germination energy of the malting barley. The current finding is in line with result of Nure [57], who revealed that the germination energy of the seed is highly responded to mineral N fertilization rates. The study also showed that the maximum germination energy was obtained at highest at application rate of 225 kg ha^-1^ urea fertilizer, while the lowest germination energy obtained from the seeds without nitrogen application.

### Correlation analysis of quality parameters on malt barley

The observed negative correlation between grain protein content and malt extract yield shows that higher protein content in grain is related to lower extract values. This is due to the fact that increased protein accumulation can decrease the availability of carbohydrate (starch) concentrations and thus result in low malt extract yield or values and also limit enzymatic hydrolysis during the malting process. This result agrees with the finding of Fox [64], who revealed that the malt extract yield or quality is affected by different environmental conditions such as temperature, soil moisture, fertilization, and available nitrogen. These factors do not directly affect extract yield; they affect the content and compositions of parameters, particularly the balance of protein content and starch, which in turn governs extract production. Similarly, the grain protein content showed a negative correlation with malt extract yield (r = −0.86 and r = −0.838), respectively [65]. Moreover, the malt extract yield was significantly negatively correlated with grain protein content [65]. The malt extract yield of malt barley grain was positively correlated with malt friability (r = 0.7) [53].

## Conclusion

Malt barley is one of Ethiopia’s most important cereal crops. It is mostly grown in the highlands area, where it is a major part of the expanding local brewing industry and serve as a source of income for smallholder farming livelihoods. Despite this advantage, soil fertility decline, limited application of organic amendments, and poor agronomic practices have adversely influenced the productivity and malt quality. This experiment revealed that a balanced use of organic compost and chemical nitrogen fertilization rates has a significant influenced on thousand-seed weight, malt extract yield, grain protein concentration, malt beta-glucan, germination capacity, and germination energy. Moreover, the integrated and interactive effects of mineral nitrogen, compost, and growing season or year influenced various malt quality parameters.

Most notably, the results clearly show that a moderate application of 69 kg N ha ⁻ ¹ mixed with 5 t ha ⁻ ¹ compost provides an optimal balance of malt quality traits, fulfilling acceptable industry standards without excessive protein content or a lower malt extract yield. This integrated management approach improves malt quality while supporting soil fertility and long-term production sustainability and offers a practical and scalable approach for smallholder farmers in the Ethiopian highlands.

The practical implication of this experiment extends beyond the experimental plots. For smallholder farmers’ in the Ethiopian highlands, the optimum fertilizer use of mineral nitrogen with compost combinations provides a viable approach to enhance malt quality traits and market value (enhancing farmers’ industry linkage) while sustaining soil health and quality. For breweries, it ensures a reliable supply of malt barley grain with high quality, which is critical for continuous and efficient malting and brewing, and also a solution for reducing malt-import dependence. The finding is also emphasize on the importance of integrating organic and chemical fertilization to improve agricultural productivity while guaranteeing long-term environmental sustainability for policymakers and developmental programs. For stronger and widely applicable recommendations, future studies conducted across multiple seasons and locations, supported by more quality parameters evaluations, are advisable.

## References

[1] Schmid K, Kilian B, Russell J. Barley domestication, adaptation and population genomics. Compendium of Plant Genomes. Springer International Publishing. 2018. p. 317–36. doi: 10.1007/978-3-319-92528-8_17

[2] Badr A, Müller K, Schäfer-Pregl R, El Rabey H, Effgen S, Ibrahim HH, et al. On the origin and domestication history of Barley (Hordeum vulgare). Mol Biol Evol. 2000;17(4):499–510. doi: 10.1093/oxfordjournals.molbev.a026330 10742042

[3] FAO. FAO Statistical Databases. 2023. https://www.fao.org/faostat/en/#home

[4] Teklemariam SS, Bayissa KN, Matros A, Pillen K, Ordon F, Wehner G. The genetic diversity of Ethiopian barley genotypes in relation to their geographical origin. PLoS One. 2022;17(5):e0260422. doi: 10.1371/journal.pone.0260422 35622864 PMC9140232

[5] FAO F and AO. Food balance sheets. Food and Agriculture Organization of the United Nations; 2014.

[6] ICARDA. Improving Ethiopian Malt Barley in Ethiopia for Better Livelihoods and Economy. 2021.

[7] Demelash A, Alehegn M, Menzir A. Diversity, spatial distribution, breeding status, production level, and use dynamics analysis of food barley in Ethiopia. Advances in Agriculture. 2025;2025(1). doi: 10.1155/aia/8830375

[8] Assefa S, Shewangizaw B, Yassin KK, Getaneh L. Growth, yield components, and yield response of food barley (Hordeum vulgare L.) to the application of sulfur nutrient under balanced fertilization at North Central Highland of Ethiopia. J Crop Sci Biotechnol. 2021;24(4):461–7. doi: 10.1007/s12892-021-00094-5

[9] Terefe Z, Feyisa T, Molla E, Ejigu W. Effects of vermicompost and lime on acidic soil properties and malt barley (Hordeum Distichum L.) productivity in Mecha district, northwest Ethiopia. PLoS One. 2024;19(12):e0311914. doi: 10.1371/journal.pone.0311914 39642131 PMC11623467

[10] Asres T, Tadesse D, Wossen T, Sintayehu A. Performance Evaluation of Malt Barley: from Malting Quality and Breeding Perspective. J Crop Sci Biotechnol. 2018;21(5):451–7. doi: 10.1007/s12892-018-0199-0

[11] Workie DM, Tasew W. Adoption and intensity use of malt barley technology package by smallholder farmers in Ethiopia: A double hurdle model approach. Heliyon. 2023;9(8):e18477. doi: 10.1016/j.heliyon.2023.e18477 37534005 PMC10392096

[12] Rani H, Bhardwaj RD. Quality attributes for barley malt: “The backbone of beer”. J Food Sci. 2021;86(8):3322–40. doi: 10.1111/1750-3841.15858 34287897

[13] Assefa A, Girmay G, Alemayehu T, Lakew A. Performance evaluation of malt barley (*Hordeum vulgare* L.) varieties for yield and quality traits in Eastern Amhara regional state, Ethiopia. Advances in Agriculture. 2021;2021:1–5. doi: 10.1155/2021/5566381

[14] Shimelis F, Mulatu Z. Effect of nitrogen fertilizer rate on grain yield and malt quality of three malt barley (<;i>;Hordeum vulgare <;/i>;L.) Varieties at Arsi Zone, Ethiopia. JPS. 2021;9(4):170. doi: 10.11648/j.jps.20210904.16

[15] Kaur AS, Yadav AK, Prakash R, Singh V. Optimum nitrogen dose and malt quality of barley varieties under saline water irrigation. ASD. 2024;(Of). doi: 10.18805/ag.d-6037

[16] Sainju UM. Reduced nitrogen rate sustains malt barley yield and quality in malt barley‐pea rotation. Agronomy Journal. 2024;116(6):3021–32. doi: 10.1002/agj2.21717

[17] Cammarano D, Holland J, Gianinetti A, Baronchelli M, Ronga D. Impact of nitrogen and water on barley grain yield and malting quality. J Soil Sci Plant Nutr. 2024;24(4):6718–30. doi: 10.1007/s42729-024-01999-0

[18] Kassie M, Tesfaye K. Influence of variety and nitrogen fertilizer on productivity and trait association of malting barley. Journal of Plant Nutrition. 2019;42(10):1254–67. doi: 10.1080/01904167.2019.1605380

[19] Tadesse K, Habte D, Admasu W, Admasu A, Abdulkadir B, Tadesse A, et al. Effects of preceding crops and nitrogen fertilizer on the productivity and quality of malting barley in tropical environment. Heliyon. 2021;7(5):e07093. doi: 10.1016/j.heliyon.2021.e07093 34095585 PMC8167224

[20] Reussi Calvo NI, Carciochi WD, Prystupa P, Queirolo I, Sainz Rozas HR. Economic optimum nitrogen rate analysis for feed and malting barley. Crop Science. 2022;62(5):1997–2010. doi: 10.1002/csc2.20808

[21] Goettl B, DeSutter T, Bu H, Wick A, Franzen D. Managing nitrogen to promote quality and profitability of North Dakota two‐row malting barley. Agronomy Journal. 2024;116(2):719–26. doi: 10.1002/agj2.21538

[22] Balducci E, Beccari G, Orfei M, Tini F, Covarelli L, Benincasa P. Nitrogen fertilization management and seeding density differently affect net blotch incidence and grain yield in one two-row and one six-row cultivar of barley. Italian Journal of Agronomy. 2024;19(3):100019. doi: 10.1016/j.ijagro.2024.100019

[23] Fang Y, Zhang X, Xue D. Genetic analysis and molecular breeding applications of malting quality QTLs in barley. Front Genet. 2019;10:352. doi: 10.3389/fgene.2019.00352 31068969 PMC6491634

[24] Rogers CW, Dari B, Neibling H, Walling J. Barley yield and malt characteristics as affected by nitrogen and final irrigation timing. Agronomy Journal. 2022;114(2):1461–74. doi: 10.1002/agj2.21036

[25] Chavarekar S, Thakral SK, Meena RK. Effect of organic and inorganic nitrogen fertilizers on quality of barley (Hordeum vulgare L.). Ann Agric Res New Series. 2013.

[26] Jaques LBA, Carvalho IR, Szareski VJ, Rodrigues HE, Dubal ÍTP, Troyjack C, et al. Physiologic quality and biochemical characters of barley seeds produced under nitrogen doses and growing environments. JAS. 2019;11(12):65. doi: 10.5539/jas.v11n12p65

[27] Pahalvi HN, Rafiya L, Rashid S, Nisar B, Kamili AN. Chemical Fertilizers and Their Impact on Soil Health. Microbiota and Biofertilizers, Vol 2. Springer International Publishing; 2021. p. 1–20. doi: 10.1007/978-3-030-61010-4_1

[28] Zhang J, He W, Wei Z, Chen Y, Gao W. Integrating green manure and fertilizer reduction strategies to enhance soil carbon sequestration and crop yield: evidence from a two-season pot experiment. Front Sustain Food Syst. 2025;8. doi: 10.3389/fsufs.2024.1514409

[29] Agegnehu G, Lakew B, Nelson PN. Cropping sequence and nitrogen fertilizer effects on the productivity and quality of malting barley and soil fertility in the Ethiopian highlands. Archives of Agronomy and Soil Science. 2014;60(9):1261–75. doi: 10.1080/03650340.2014.881474

[30] Oueriemmi H, Kidd P, Trasar-Cepeda C, Rodríguez-Garrido B, Zoghlami R, Ardhaoui K, et al. Evaluation of composted organic wastes and farmyard manure for improving fertility of poor sandy soils in Arid Regions. Agriculture. 2021;11(5):415. doi: 10.3390/agriculture11050415

[31] Ghouili E, Abid G, Hogue R, Jeanne T, D’Astous-Pagé J, Sassi K, et al. Date palm waste compost application increases soil microbial community diversity in a cropping barley (*Hordeum vulgare* L.) field. Biology (Basel). 2023;12(4):546. doi: 10.3390/biology12040546 37106747 PMC10135526

[32] Agegnehu G, Nelson PN, Bird MI. The effects of biochar, compost and their mixture and nitrogen fertilizer on yield and nitrogen use efficiency of barley grown on a Nitisol in the highlands of Ethiopia. Sci Total Environ. 2016;569–570:869–79. doi: 10.1016/j.scitotenv.2016.05.033 27288288

[33] Mengistu T, Gebrekidan H, Kibret K, Woldetsadik K, Shimelis B, Yadav H. The integrated use of excreta-based vermicompost and inorganic NP fertilizer on tomato (*Solanum lycopersicum* L.) fruit yield, quality and soil fertility. Int J Recycl Org Waste Agricult. 2017;6(1):63–77. doi: 10.1007/s40093-017-0153-y

[34] Yimer AH. Influence of organic fertilizers on productivity of barley: a review. ASD. 2021;(Of). doi: 10.18805/ag.df-374

[35] Zhou Z, Zhang S, Jiang N, Xiu W, Zhao J, Yang D. Effects of organic fertilizer incorporation practices on crops yield, soil quality, and soil fauna feeding activity in the wheat-maize rotation system. Front Environ Sci. 2022;10. doi: 10.3389/fenvs.2022.1058071

[36] Muluneh MW, Talema GA, Abebe KB, Dejen Tsegaw B, Kassaw MA, Teka Mebrat A. Determinants of organic fertilizers utilization among smallholder farmers in south gondar zone, Ethiopia. Environ Health Insights. 2022;16:11786302221075448. doi: 10.1177/11786302221075448 35140472 PMC8819767

[37] Lu W, Zhou Y, Ma X, Gao J, Guo J, Fan X, et al. Impacts of organic fertilizer substitution on soil ecosystem functions: synergistic effects of nutrients, enzyme activities, and microbial communities. Agronomy. 2025;15(12):2798. doi: 10.3390/agronomy15122798

[38] Shaji H, Chandran V, Mathew L. Organic fertilizers as a route to controlled release of nutrients. Controlled Release Fertilizers for Sustainable Agriculture. Elsevier; 2021. p. 231–45. doi: 10.1016/b978-0-12-819555-0.00013-3

[39] Gomez KA, Gomez AA. Statistical Procedures for Agricultural Research. Second Edition ed. Los Blaw, Philippines: John Wiley & Sons, Inc; 1984.

[40] Kassie M, Tesfaye K. Malting barley grain quality and yield response to nitrogen fertilization in the arsi highlands of Ethiopia. J Crop Sci Biotechnol. 2019;22(3):225–34. doi: 10.1007/s12892-019-0080-0

[41] Azam MF, Bayar J, Iqbal B, Ahmad U, Okla MK, Ali N, et al. Planting pattern and nitrogen management strategies: positive effect on yield and quality attributes of Triticum aestivum L. crop. BMC Plant Biol. 2024;24(1):845. doi: 10.1186/s12870-024-05537-z 39251892 PMC11382503

[42] Boulelouah N, Berbache M, Bedjaoui H, Selama N, Rebouh N. Influence of Nitrogen fertilizer rate on yield, grain quality and nitrogen use efficiency of durum wheat (*Triticum durum Desf*) under algerian semiarid conditions. Agriculture. 2022;12(11):1937. doi: 10.3390/agriculture12111937

[43] Omirou M, Fasoula D, Stylianou M, Zorpas AA, Ioannides IM. N-source determines barley productivity, nutrient accumulation, and grain quality in cyprus rainfed agricultural systems. Int J Environ Res Public Health. 2023;20(5):3943. doi: 10.3390/ijerph20053943 36900954 PMC10001598

[44] Bahmani M, Juhász A, Broadbent J, Bose U, Nye-Wood MG, Edwards IB, et al. Proteome phenotypes discriminate the growing location and malting traits in field-grown barley. J Agric Food Chem. 2022;70(34):10680–91. doi: 10.1021/acs.jafc.2c03816 35981222 PMC9449971

[45] Edney MJ, O’Donovan JT, Turkington TK, Clayton GW, McKenzie R, Juskiw P, et al. Effects of seeding rate, nitrogen rate and cultivar on barley malt quality. J Sci Food Agric. 2012;92(13):2672–8. doi: 10.1002/jsfa.5687 22523006

[46] Shilev S, Mitkov A, Popova V, Neykova I, Minev N, Szulc W, et al. Fertilization type differentially affects barley grain yield and nutrient content, soil and microbial properties. Microorganisms. 2024;12(7):1447. doi: 10.3390/microorganisms12071447 39065216 PMC11279231

[47] Tadesse K, Abdulkadir B, Admasu W, Habte D, Admasu A, Tadesse A, et al. Soil test-based phosphorus fertilizer recommendation for malting barley production on Nitisols. Open Agriculture. 2022;7(1):171–80. doi: 10.1515/opag-2022-0078

[48] Halstead M, Morrissy C, Fisk S, Fox G, Hayes P, Carrijo D. Barley grain protein is influenced by genotype, environment, and nitrogen management and is the major driver of malting quality. Crop Science. 2022;63(1):115–27. doi: 10.1002/csc2.20842

[49] Habiyaremye C, Schroeder KL, Reganold JP, White D, Packer D, Murphy KM. Effect of Nitrogen and Seeding Rate on β-Glucan, Protein, and Grain Yield of Naked Food Barley in No-Till Cropping Systems in the Palouse Region of the Pacific Northwest. Front Sustain Food Syst. 2021;5. doi: 10.3389/fsufs.2021.663445

[50] Tadesse K, Mekonnen A, Admasu A, Admasu W, Habte D, Tadesse A, et al. Malting barley response to integrated organic and mineral nutrient sources in Nitisol. Int J Recycl Org Waste Agricult. 2018;7(2):125–34. doi: 10.1007/s40093-018-0198-6

[51] Alotaibi MM, Aljuaid A, Alsudays IM, Aloufi AS, AlBalawi AN, Alasmari A, et al. Effect of Bio-Fertilizer Application on Agronomic Traits, Yield, and Nutrient Uptake of Barley (Hordeum vulgare) in Saline Soil. Plants (Basel). 2024;13(7):951. doi: 10.3390/plants13070951 38611480 PMC11013266

[52] EQSA. Malting Barley Specification. Addis Ababa, Ethiopia: EQSA; 2006.

[53] Kumar D, Sharma AK, Narwal S, Sheoran S, Verma RPS, Singh GP. Utilization of grain physical and biochemical traits to predict malting quality of barley (*Hordeum vulgare* L.) under sub-tropical climate. Foods. 2022;11(21):3403. doi: 10.3390/foods11213403 36360015 PMC9657330

[54] Khaleghdoust B, Esmaeilzadeh-Salestani K, Korge M, Alaru M, Möll K, Värnik R, et al. Barley and wheat beta-glucan content influenced by weather, fertilization, and genotype. Front Sustain Food Syst. 2024;7. doi: 10.3389/fsufs.2023.1326716

[55] Kristó I, Vályi-Nagy M, Rácz A, Irmes K, Szentpéteri L, Jolánkai M, et al. Effects of nutrient supply and seed size on germination parameters and yield in the next crop year of winter wheat (*Triticum aestivum* L.). Agriculture. 2023;13(2):419. doi: 10.3390/agriculture13020419

[56] Zhong M, Miao Y, Han S, Wang D. Nitrogen addition decreases seed germination in a temperate steppe. Ecol Evol. 2019;9(15):8441–9. doi: 10.1002/ece3.5151 31410252 PMC6686302

[57] Nure K. Effect of seed sources and rates of nitrogen fertilizer on seed quality, yield and yield related traits of malt barley (*Hordeum vulgare* L.) at Kulumsa, Arsi Zone, Ethiopia. Haramaya University. 2023.

[58] EBC (European Brewery Convention). Analytical European Brewery Convention. Carl, Getranke-Fachver; 1998.

[59] Mills AAS, Izydorczyk M, Choo TM A, Durand J, Mountain N, Sorrells M, et al. Cultural practices to improve malt barley quality in the northeast with focus on the craft sector. Can J Plant Sci. 2021;101(1):39–52. doi: 10.1139/cjps-2020-0011

[60] Adeyemi OE. Barley yield and protein response to nitrogen and sulfur fertilizer rates and application timing. University of Idaho; 2023.

[61] Saygili I. Barley yield and malt quality affected by fall and spring planting under rainfed conditions. PeerJ. 2023;11:e15802. doi: 10.7717/peerj.15802 37601258 PMC10434083

[62] Macleod A, Izydorczyk MS, O’donovan JT. Effect of increasing nitrogen fertilization on barley protein content and endosperm modification during malting. 2015.

[63] Parashar A, Sharma S, Parashar K, Dogra P, Kumawat C, Patra A, et al. Yield and malt quality of barley (Hordeum vulgare) impacted by nitrogen and sulphur application. Indian J Agri Sci. 2022;91(12):1783–7. doi: 10.56093/ijas.v91i12.120807

[64] Fox GP. Chemical composition in barley grains and malt quality. Advanced Topics in Science and Technology in China. Springer Berlin Heidelberg; 2009. p. 63–98. doi: 10.1007/978-3-642-01279-2_3

[65] Cai S, Yu G, Chen X, Huang Y, Jiang X, Zhang G, et al. Grain protein content variation and its association analysis in barley. BMC Plant Biol. 2013;13:35. doi: 10.1186/1471-2229-13-35 23452582 PMC3608362

